# Spatially governed climate factors dominate management in determining the quantity and distribution of soil organic carbon in dryland agricultural systems

**DOI:** 10.1038/srep31468

**Published:** 2016-08-17

**Authors:** Frances C. Hoyle, Rebecca A. O’Leary, Daniel V. Murphy

**Affiliations:** 1 Department of Agriculture and Food Western Australia, 3 Baron-Hay Court, South Perth, WA 6151, Australia; 2 Soil Biology and Molecular Ecology Group, School of Earth and Environment, Institute of Agriculture, Faculty of Science, The University of Western Australia, 35 Stirling Highway, Crawley, WA 6009, Australia

## Abstract

Few studies describe the primary drivers influencing soil organic carbon (SOC) stocks and the distribution of carbon (C) fractions in agricultural systems from semi-arid regions; yet these soils comprise one fifth of the global land area. Here we identified the primary drivers for changes in total SOC and associated particulate (POC), humus (HOC) and resistant (ROC) organic C fractions for 1347 sample points in the semi-arid agricultural region of Western Australia. Total SOC stock (0–0.3 m) varied from 4 to 209 t C ha^−1^ with 79% of variation explained by measured variables. The proportion of C in POC, HOC and ROC fractions averaged 28%, 45% and 27% respectively. Climate (43%) and land management practices (32%) had the largest relative influence on variation in total SOC. Carbon accumulation was constrained where average daily temperature was above 17.2 °C and annual rainfall below 450 mm, representing approximately 42% of the 197,300 km^2^ agricultural region. As such large proportions of this region are not suited to C sequestration strategies. For the remainder of the region a strong influence of management practices on SOC indicate opportunities for C sequestration strategies associated with incorporation of longer pasture phases and adequate fertilisation.

Agricultural soils contribute to the balance of greenhouse gas emissions as both a sink and source, as well as being vital in securing global food supplies from a limited land base. Worldwide, carbon (C) emissions resulting from the depletion of soil organic matter pools are estimated at 66 to 90 Pg C^1^. Semi-arid soils constitute nearly one fifth of the global land area^2^ and are widely used for agricultural production. Historical clearing of native vegetation and subsequent cultivation of Australian semi-arid dryland cropping regions has resulted in substantial losses of organic C from soils^1^,^3^ that are likely to reflect global C losses (quantified at 42 to 59%)^4^. Subsequently practices including a transition from pasture phases to more intensive cropping^5^, continued tillage^6^, low retention of plant residues^7^ and increased soil erosion^8^ have also contributed to a continued decline in soil organic carbon (SOC).

While increasing C storage in soils has been proposed as a mechanism to moderate greenhouse gas emissions, production based benefits including enhanced nutrient availability, soil aggregation and soil buffering may also be realised. This potential exists where long-term use of land management practices can be adopted that restore and maintain C; such as higher net primary productivity, residue retention and low soil disturbance (no tillage). Thus managing soils to increase the amount and persistence of organic C could be considered a win-win strategy for agricultural systems in mitigating climate change and securing food production.

Semi-arid areas are suggested to be the most sensitive to global warming and most likely to reflect rapid climate change^9^. Climate variables including rainfall and temperature^10^,^11^ often account for more of the variation in soil C stocks when compared with land use and management^11^. Therefore understanding the effects of climate change on C storage is critical^12^. The south-western Australian grain-growing region consists of 197,300 km^2^ (19 million ha) of semi-arid land^13^, exporting up to 80% of its coarse grains, making this region an important contributor in feeding the growing world population. With Western Australia having one third of the arable land area in Australia, it is estimated to contain more SOC than any other state or territory^14^. However, the south-west of Western Australia has experienced a 15% decline in mean annual rainfall since the mid-1970s^15^, and a further 5 to 11% decline is projected by 2050 relative to pre-1990 levels, with most decreases likely to be experienced during the winter months^16^. Since the mid-1970s annual average temperatures are also expected to increase in the order of 0.5 to 1.5 °C in line with global average temperatures during the same period^17^. Given current climate change projections for the south-west of Western Australia a decrease in wheat production of approximately 13% by 2050 is anticipated, with similar outcomes for sheep production^17^. As such it is necessary to determine how to best maintain and where possible increase existing SOC stocks under a changing climate. Climate-induced land use distribution is also likely to change, with cereal based cropping in the marginal rainfall regions being abandoned and increased cropping opportunities displacing pasture for animal production in high rainfall regions.

In combating potential drying scenarios, managing limitations to available water is critical to the continued viability of the agricultural sector and maintenance of SOC. The soils of Western Australia are ancient and highly weathered^18^, having poor water and soil buffering capacity as a consequence. Nearly 6 million hectares within the agricultural zone of the south-west of Western Australia is classed as having multiple constraints to plant production^19^. These include soil-borne diseases, low soil pH, water repellence, limited soil water storage capacity, subsurface compaction, erosion and in higher rainfall zones the occurrence of water-logging. Water use and resultant yield gaps vary by site, season and management. Average regional water use efficiency by grain crops from 1996 to 2008 for the Western Australian agricultural region is reported at 56% (range 49.8 to 63.0%) of the rainfall limited yield potential^20^. With current wheat crops yielding 1.60 t ha^−1^ on average (2000 to 2013)^13^ the calculated theoretical rainfall limited yield potential is *ca*. 2.84 t ha^−1^, a yield gap of 1.24 t ha^−1^.

Changes in net primary productivity inputs and resultant changes in soil organic matter are regulated in part by the most limiting factor or scarcest resource. Hence the influence of factors such as source and timing of fertiliser, soil disturbance, as well as biological and physio-chemical constraints on water and nutrient availability that influence resource use efficiency limit both net primary productivity and potentially C storage. Kirkby *et al.*^21^ propose a requirement for a near constant C: nitrogen (N): phosphorus (P): sulphur (S) ratio in stable soil C pools; this implies that in soils with inherently low soil fertility the potential for C storage is limited by nutrient deficiencies. In Australian cropping soils typical fertiliser use efficiency has been estimated at between 30 and 50% of applied N^22^, between 20 and 50% of applied S^22^ and between 12% and 70%^23^ for P in alkaline soils dependent on method of determination^24^,^25^, with many of the afore mentioned factors influencing uptake. In dairy systems Gourley *et al.*^26^ noted efficiency of use for N was 26%, P 35%, and S 21% when considering a whole-farm nutrient balance. As such it is likely that rapid loss of nutrients combined with poor availability are constraining soil C storage.

We hypothesise that lower than maximal net primary productivity (due to land management choices and soil constraints to plant growth) has limited SOC sequestration to a greater extent than the potential decrease in rainfall that may be realised under climate change scenarios. This is particularly evident in that although average rainfall has decreased by up to 20% over the past decade in Western Australia, there has been little influence on net primary productivity^27^. Asseng & Pannell^28^ suggest this is often linked to a lower frequency of very wet years, a decline in rainfall during non-critical growth stages not associated with plant establishment or grain filling, and inherent soil constraints on root growth and water limited potential yield which often result in less than 60% of available water being used. This suggests that more efficient exploitation of soil resources including water and nutrients to increase net primary productivity can buffer against losses imposed by climate restrictions, and is in fact a much larger concern in terms of restricting plant residue inputs to the soil in building SOC. As such this study aimed to i) quantify the influence of climate, soil parameters and land management practices on total SOC stocks and associated organic C fractions in Western Australian agricultural soils; and ii) identify the extent of this semi-arid agricultural region where future increases in SOC are unlikely to occur as a result of climate change and associated shifts in land use.

## Results

### Total soil organic carbon

**Figure 1:**
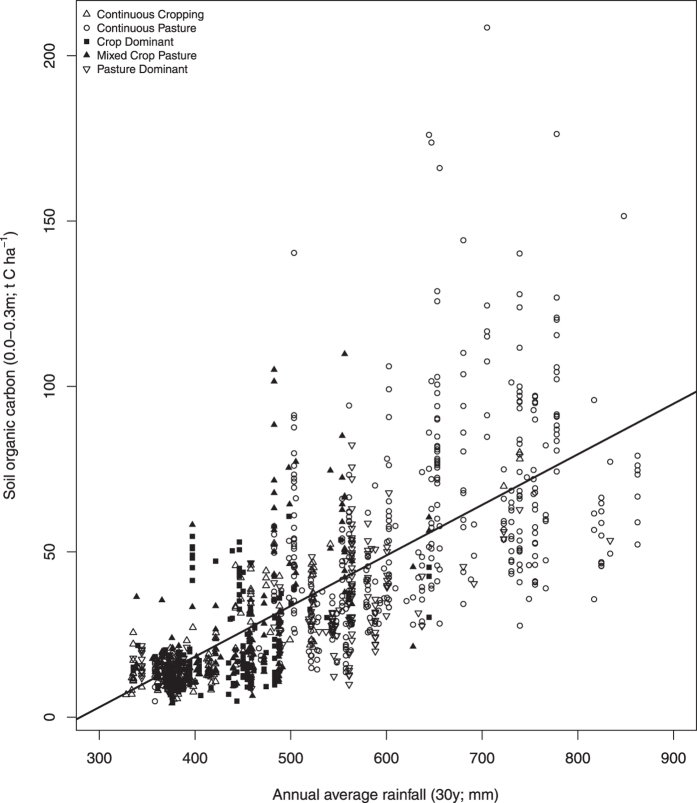
Total soil organic carbon (t C ha^−1^; 0–0.3 m) and annual average rainfall (mm) based on 30 year history for sites sampled in Western Australia (n = 1348 sample points). Symbols represent different land sequences as depicted in the legend. The solid line represents the regression analysis (p < 0.0001, R^2^ = 0.54).

The mass of total SOC at individual sites ranged from 1.5 to 147.6 t C ha^−1^ (mean 21.5 t C ha^−1^) in the 0–0.1 m layer, and from 4.2 to 208.5 t C ha^−1^ (mean 34.5 t C ha^−1^) in the 0–0.3 m soil layer (see Supplementary Table S1). Nearly two thirds (61%) of SOC from the 0–0.3 m layer was located in the top 0.1 m soil. Between 72% (0–0.1 m) and 79% (0–0.3 m) of the variability in total SOC stocks could be attributed to measured soil, climate and land management parameters, with climate variables (rainfall and temperature) demonstrating the greatest importance (P < 0.001; Table 1a,b). Regardless of land sequence, total SOC stocks generally increased with rainfall (Fig. 1). Splitting rules based on predictor variables for binary regression trees determined that SOC (0.0–0.1 m) differed at sites with <473 mm, 473 to 600 mm and >600 mm rainfall (Table 1a, see Supplementary Fig. S1), and for 0.0 to 0.3 m depth site differences were above and below 600 mm (Table 1b, see Supplementary Fig. S2). At sites with <600 mm rainfall a secondary split based on temperatures above or below 17.2 °C was also determined (Table 1b, see Supplementary Fig. S2).

Removing climate parameters resulted in considerably less variability being explained for changes in SOC and generally strengthened the relative influence of management, soil textural group (for sites <600 mm) and soil pH (Table 1a,b). Rotation, soil textural group, stock (presence of animals) and the amount of S fertiliser applied were included in the final model across all data but of lesser importance compared to climate variables. Vapour pressure deficit (VPD30y) showed high relative importance but was strongly co-correlated to temperature (R^2^ = 0.96) and as such was not included in the final model.

In high rainfall areas (>600 mm; 0–0.3 m; n = 302) the apparent influence of historical S fertiliser application on SOC was associated with a dominance of pasture and permanent pasture systems and higher net primary productivity. Sites where average annual rainfall (mm) was >600 mm (n = 302) had nearly three times the amount of total SOC to 0.1 m (44 t C ha^−1^) and 0.3 m (68 t C ha^−1^) than those <600 mm (n = 1046, 15 and 25 t C ha^−1^ respectively).

### Temperature

**Figure 2:**
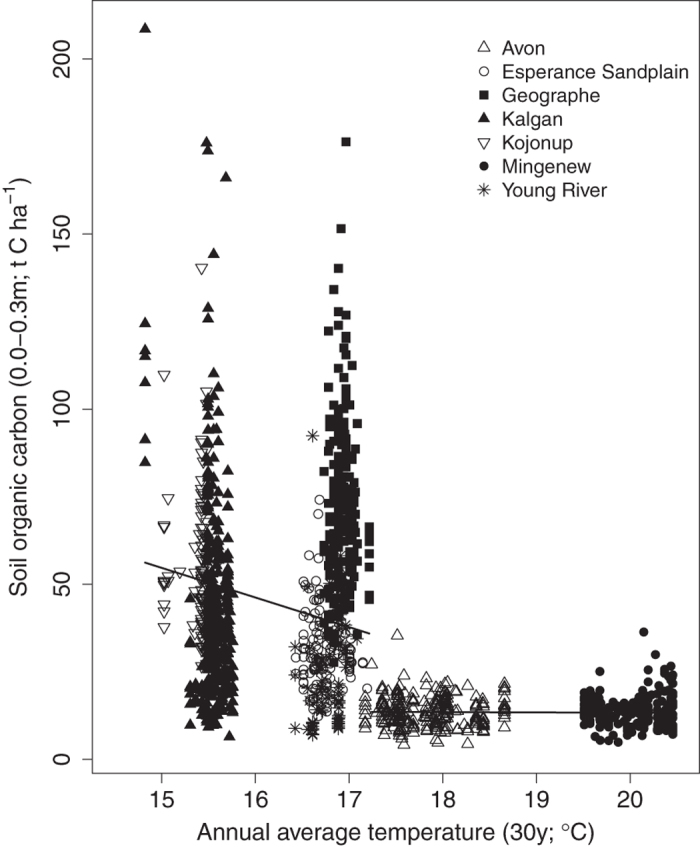
Total soil organic carbon (t C ha^−1^; 0–0.3 m) in Western Australia (n = 1348 sample points) against average annual daily temperature (°C) based on 30 year history. Symbols represent different regions as depicted in the legend. The solid line represents the change point regression analysis (see Table 2).

The influence of temperature was significant (P < 0.0001, Fig. 2) for SOC stocks at both depth intervals. Sites with an average daily temperature >17.2 °C over the previous 30 year period were consistently lower in total SOC (0–0.3 m), and in this instance there was a strong positive correlation between temperature and latitude (reflecting a north-south temperature gradient) for the sites sampled (R^2^ = 0.94; n = 1348). Change point regression analysis determined this decrease in total SOC was significant (p < 0.0001) for both 0–0.1 m and 0–0.3 m layers (Fig. 2). Below and above this temperature change point, linear regression was applied to explore the relationship between the total amount of SOC and temperature at 0–0.3 m (Fig. 2, Table 2). Increasing temperature was negatively correlated (R^2^ = −0.51) with total SOC. At sites where SOC was constrained by temperature >17.2 °C there was representation from all land sequences, soil groups and rainfall and thus not considered to be a co-correlation to another variable. At sites with <600 mm of annual rainfall and average daily temperature >17.2 °C (n = 497), SOC stocks (0–0.3 m) were considerably lower (13.4 t C ha^−1^) compared to cooler environments (n = 549, 35.1 t C ha^−1^). At high rainfall (>600 mm) sites, no change in SOC stocks was associated with temperature.

### Soil pH

**Figure 3:**
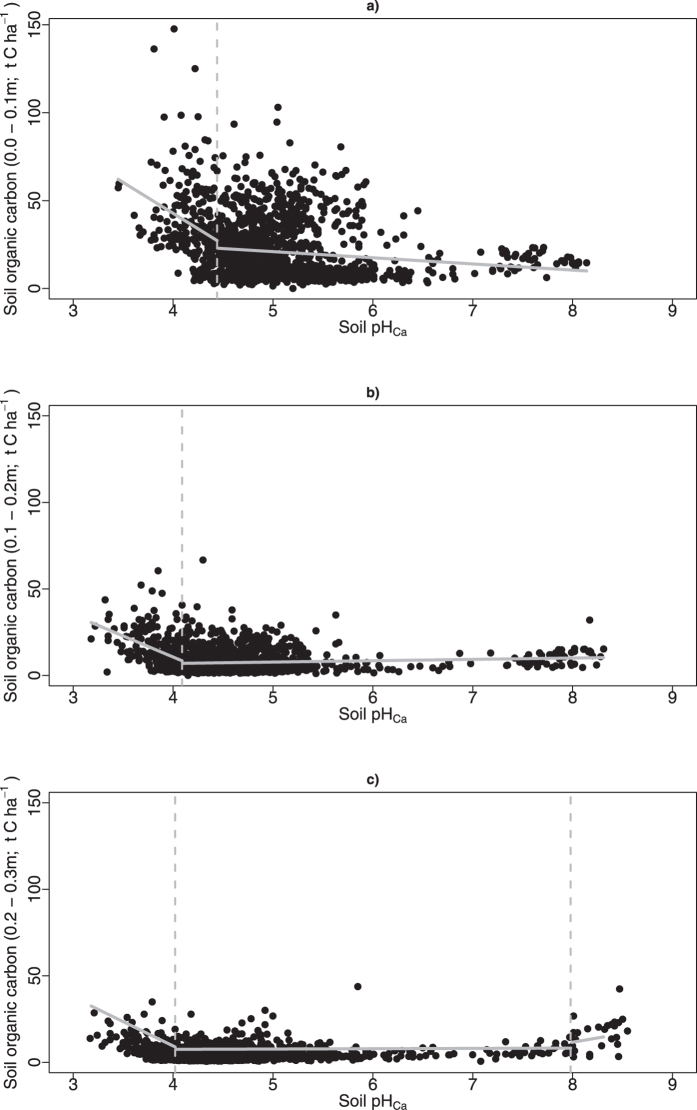
Total soil organic carbon (t C ha^−1^) in soils from (**a**) 0–0.1 m, (**b**) 0.1–0.2 m and (**c**) 0.2–0.3 m for 1348 sample points in Western Australia against soil pH. The black circles are the observed data, grey vertical dash lines are the change points and grey solid lines are the regressions applied to below and above the change point.

Change point regression analysis (n = 1348) identified a significant (p < 0.0001) change in soil pH associated with the total amount of SOC at each depth interval (Fig. 3a–c; Table 3), with temperature and on-farm management contributing to variation. For all depths combined when soil pH was >4.6, there was a significant (F = 74.924; p < 0.0001) decline in SOC compared to more acid sites (Table 3). As soil depth increased the change point for pH became more acidic and the relative influence of pH on fractions of C increased (Table 3). Higher total SOC stocks were observed above a pH of 7.9 but the site number is limited (n = 14) at this range and represents a single region and depth profile (Fig. 3, Table 3).

### Soil organic carbon fractions

Assuming all C could be allocated based on proportional values for different SOC fractions obtained by mid-infrared (p = 0.05, n = 1414), the attribution of organic C to particulate, humus and resistant C fractions at 0.0–0.1 m across all sites averaged 32% (6.0 t C ha^−1^), 42% (7.7 t C ha^−1^) and 26% (4.8 t C ha^−1^) respectively. At 0.0–0.3 m depth, the proportion of particulate C declined to 28% (9.3 t C ha^−1^), humus increased to 45% (14.7 t C ha^−1^) and resistant fraction remained unchanged at 27% (8.7 t C ha^−1^). However, as to be expected a wide variation in fractional values existed within the data set (see Supplementary Table S3 and Fig. S2). Between 72 and 83% of variability within the SOC fractions could be explained by factors included in the analysis (Table 4a,b), soil textural group and pH below 0.1 m depth were of greater relative importance than was evident for total SOC (Table 1a,b).

The relative importance of model coefficients and smoothing terms suggest that humus and resistant fractions (0.0–0.3 m) are more closely linked to soil texture than the particulate fraction (Table 4b). When climate variables are removed from the analysis soil textural group, soil pH and fertiliser application make up a considerable component of the variability in the size of the humus fraction.

Forward regression analysis suggests a stronger relative influence of soil textural group on the resistant fraction (0.0–0.3 m), and reflects differences measured in total SOC. Where climate is excluded from the tree analysis, 59% of the variability can be explained by soil pH, crop rotation, soil textural group and fertiliser influences. A stronger climate influence in surface soil to 0.1 m is evident and when removed soil textural group, soil pH and management influences explain between 28 and 63% of the variability.

## Discussion

Factors such as (i) the spatial variability associated with measurement of SOC stocks^14^,^29^,^30^, (ii) large land areas in Western Australia where little or no soil C data exists^31^, and (iii) a poor understanding of the relative influence of climate and land management variables on SOC stocks has contributed to creating barriers to successful landholder engagement in soil C sequestration. This is despite soil organic matter being widely regarded as critical to soil function and plant productivity. Our findings indicate that increasing SOC was associated with higher annual rainfall, lower temperature and greater frequency of pastures indicating enhanced C storage associated with longer growing season and higher net primary productivity potential. This appeared relatively independent of any soil textural group influence.

### Climate regulators of soil organic carbon

**Figure 4:**
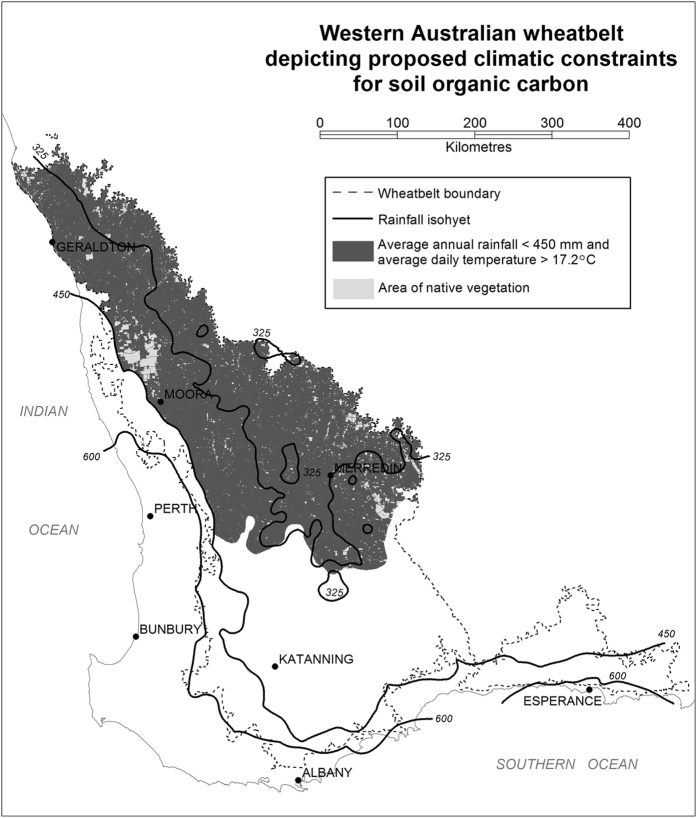
Map of Western Australian indicating areas of the agricultural region (wheatbelt) with annual average rainfall <450 mm and temperature >17.2 °C. Map generated from data provided by the State of Queensland (Department of Environment and Resource Management). This image was generated using GeoMedia^®^, which is a software product owned by Intergraph Corporation doing business as Hexagon Geospatial. ©2011–2016 Hexagon AB and/or its subsidiaries and affiliates. (http://www.hexagongeospatial.com/products/producer-suite/geomedia).

Annual rainfall was a primary regulator of surface SOC (0–0.1 m) levels in Western Australia, with regression tree splits evident at 473 and 600 mm. A similar split was also identified for deeper SOC stocks (0–0.3 m) at 602 mm. The significant (p < 0.0001) decline in total SOC (0–0.1 m) with average annual daily temperature >17.2 °C and annual rainfall <450 mm suggests a critical limit for sites in storing SOC for this semi-arid region. Regression trees show that at sites where average daily temperature is <17.2 °C, sites ≥551 mm rainfall stored more C to 0.3 m than those <551 mm. This supports previous observations^32^ in other regions of Australia which determined a critical temperature of >17.4 °C. When applied to the Western Australian agricultural region this equates to over 8 M ha of semi-arid arable land (Fig. 4) potentially having a constraint to SOC accumulation due to climate.

Current predictions for changes in crop yields with climate change are based on the performance of current farming systems under relative changes in rainfall and temperature. Van Gool^33^ reports 43% of the Western Australian cropping region is likely to experience a decline in potential wheat yield of between 10 and 30%, with less than 10% of areas (those closest to the coast under highest rainfall) predicted to increase. Increasing the storage and utilisation of plant available water and higher C assimilation by plants^34^ may combat this to some extent. While it is not possible to alter rainfall for a site, it is important to recognise that many areas of the Western Australian agricultural region do not achieve their rainfall limited yield potential^35^. This suggests that optimising plant access to soil water through better infiltration and identifying/removing soil constraints to plant growth could increase yields within the context of a changing climate. This could be achieved by ameliorating and managing soils to increase water entry into soil and the capacity to store water to depth, as well as promoting early vigour and better plant growth through the management of ‘hostile’ subsoils to improve effective rooting depth for better water capture. In Western Australia, managing water repellent topsoils and soil acidity are primary strategies to achieve this.

### Land management regulators of soil organic carbon

The influence of land management on SOC stocks was similarly dependent on climate and soil conditions at any given location as previously determined for Eastern Australia^36^. Notwithstanding climate influences, land management practices that remove constraints to plant growth and optimise organic matter inputs to increase soil C storage should be considered where it is cost effective to do so^37^. Increasingly, pasture dominated landscapes are transitioning to grain cropping with the advent of (and future predictions for) a warmer, drier climate in the southwest of Western Australia. Together with historically falling profitability of animal enterprises this has resulted in increasing interest in cropping enterprises particularly in the 450 to 550 mm rainfall zone^38^. Soil C loss associated with the transition from pasture to cropping has been determined on average at 59%^5^ and suggests a larger impact in intermediate rainfall areas (400–500 mm annual rainfall) of up to 75%. Constraining climate drivers and changes in land sequence from pasture to crop dominant systems are likely to negatively impact upon the potential for C storage in dryland farming areas and suggests future depletion of soil C in regions where a greater proportion of land is transitioning from pasture to cropping. Large variations in the rate of loss and length of time that C can be accumulated or lost exist depending on net primary productivity of the system, soil disturbance (tillage) and soil condition. Within farming systems, a practice shift to less cultivated systems and vegetation retention can decrease the risk of SOC decline^39^.

Our data set identified soil pH as a key variable that is readily managed in acid soils and often provides productivity gains sufficient to recover costs associated with application^40^. About half of Australia’s agricultural land has a surface soil pH of less than or equal to 5.5, which is below optimal for extremely acid-sensitive agricultural crops, and below the optimal level to prevent subsoil acidification^41^. In Western Australia extensive measurement of soil pH suggests 65% of topsoil samples (0.0–0.1 m, n = 161,000 samples) and 50% of subsoil samples (0.1–0.3 m, n = 67,000 samples) are below a target pH of 5.5 for the topsoil and 4.8 for the subsoil respectively^40^. Our findings indicate a build-up of SOC at sites with pH <4.5 suggesting slower turnover of organic matter fractions in very acid soils. As such ameliorating acid soils with lime to achieve a recommended target pH of 5.5^40^ (0.0–0.1 m) in order to optimise plant production, is likely to cause a permanent loss of SOC. With the increasing adoption of lime to ameliorate low pH soils (current use estimated at 1.3 million tonnes in 2013 at an average application rate of 1 t ha^−1^), the areas of agricultural land with very low pH <4.5 may be at risk of deteriorating soil organic matter. While losses of C from low pH soils could, in theory, be recovered after the application of lime by long term improvements in the soil resource condition and associated higher net primary productivity in high yielding environments; modelled changes in rainfall and temperature associated with global greenhouse gas emissions suggest this is unlikely^42^.

Nutrient status as indicated by application of fertilisers over a five year period were a third order influence on soil C stocks and were largely evident only once climate influences were removed. Soil C stocks showed a consistent positive response to S and indicates that this nutrient may limit net C sequestration. Increasing amounts of N and P fertiliser were generally associated with a decline in total soil C stocks in low and medium rainfall areas (<600 mm). Higher production zones (>600 mm) showed increased C stocks associated with highly productive pasture systems with the application of N. Humus and resistant C fractions tended to increase with nutrient fertiliser application – particularly N and S suggesting negative changes in total C were associated with the rapidly transitioning particulate C fraction. This suggests the response in stable C fractions in this study supports the C storage hypothesis proposed by Kirkby *et al.*^21^ associated with the C:N:P:S ratio in this environment, as opposed to an increase in net primary productivity which should have been evident in the particulate fraction. Therefore both the application and efficiency of use for nutrients could be expedient in addressing increased productivity^26^ and optimising potential soil C storage.

### Distribution of soil organic carbon fractions

Changes to the size or proportion of SOC allocated to different fractions can help our understanding of the effect of changes in management and climate on C dynamics^43^ and longer term stability^44^. While time consuming to physically isolate stable fractions of SOC, mid-infrared spectral analysis has been successfully used to determine allocation of C to physically differentiated pools (POC, HOC, ROC) with different turnover times^45^,^46^. In this study the allocation of C to different fractions was more strongly associated with soil textural group and pH than climate, though this remained an important contributor and most likely reflects a longer term role in the capacity of a soil to protect C from decomposition in more stable forms. This is supported by earlier work which determined the fraction associated with heavy clay and silt sized material had relatively slow turnover^45^, and in Western Australian soils included a significant amount of highly resistant and relatively inert resistant organic C (primarily charcoal)^46^. The relative importance of model coefficients and smoothing terms in this study suggests rapid turnover^47^ and loss of the particulate fraction from the system as influenced by soil textural group and pH, N and S fertiliser application, presence of animal stock and land use. Excluding climate these variables contributed to nearly 60% of the variation observed in POC. Similar to previous work^43^ our results suggest a strong influence of land management on net primary productivity (inputs) which is of primary importance in maintaining particulate organic C in soil.

## Methods

### Region description

Soils from the south-west of Western Australia were collected (n = 1160 sites; n = 1348 sample points) from within a largely level or gently undulating landscape and represent the majority of the variation in climate, soil texture^48^ and land use sequence within the agricultural region (see Supplementary Fig. S3). Deep sands and duplex soils (i.e. texture contrast with sand over clay) dominate the dataset (see Supplementary Table S2) and are some of the most ancient and infertile soils managed for agricultural production globally^49^. Perennial based beef and dairy systems dominate the high rainfall areas (>600 mm) in southern coastal regions. Rain-fed crop production systems of Western Australia are typified by 7 months of active crop growth and dominate areas with <600 mm rainfall, with livestock production enterprises often an important component of these systems.

### Site selection and soil sampling

We assessed changes in total SOC and particulate (POC), humus (HOC) and resistant (ROC) organic C fractions for all sites across five land sequences and eight soil textural groups in semi-arid dryland agricultural soils in Western Australian. Sampling sites spanned a rainfall gradient ranging from 323 to 862 mm (see Supplementary Fig. S3). Sites represented five primary land sequences as defined by the previous 10 years rotation (see Supplementary Table S2). These were continuous cropping, crop dominant (7–9 years out of 10 in crop), mixed crop and pasture (4–6 years out of 10 in crop), pasture dominant (1–3 years out of 10 in crop) and permanent pasture; with the livestock enterprise being either present or absent. Of these, 1160 sites comprised full data sets for all parameters including historical fertiliser applications (see Supplementary Fig. S3).

Soil was collected during the summer fallow (2010, 2011) while the soil is naturally field dry (<2% gravimetric water content) and could be stored without chilling^50^. This minimised potential soil organic matter decomposition during storage and decreased the likelihood of plant roots from different rotations contaminating the sample and confounding analysis for C content. Soil was collected to a depth of 0.3 m in 0.1 m increments using a hand-held sand auger (0.05 m diameter) at 10 randomly selected grid nodes as described previously^51^ and composited by depth. A pit at the southwest corner of each grid was used to classify soil according to the Australian Soil Classification (ASC^52^; http://www.clw.csiro.au/aclep/asc_re_on_line/soilkey.htm) and Western Australian soil group (WASG^48^). Soils (n = 35 soil groups) were then attributed to larger soil groups based on soil textural class to increase the number of observations in each soil group (n = 8 textural classes^48^; see Supplementary Table S2).

### Meta-data

Average annual historical rainfall (Rain30y), daily historical temperature (Temp30y) and vapour pressure deficit (VPD30y) for the 30 year period prior to sampling for each geo-referenced site was interpolated (0.05 degree accuracy; www.longpaddock.qld.gov.au/silo). Landholder records up to 10 years prior to sampling were used to determine land sequence (10y), animal stock presence (10y) and fertiliser use (5y).

### Soil analyses

Total SOC was determined for the <2 mm soil fraction by total combustion using a Vario EL Elementar analyser (Elementar Analysensysteme, Hanau, Germany) on oven-dried finely ground soil after having been acid pre-treated where required for carbonates. Soil organic C stock was calculated as the mass of organic C, over a specified area and depth of soil^53^ and was adjusted on a volumetric basis using bulk density (BD, g cm^−3^) measured using either a manual core measure or a gamma-neutron density meter^29^ and corrected for any gravel component.

A range of known soil constraints to crop production (see Supplementary Table S2) and climate variables influencing net primary productivity were measured. Soil DNA extractions were used to determine major soil-borne plant pathogens^54^. Electrical conductivity (EC) and soil pH were determined in 1:5 (v/v) soil solution extracts using water and CaCl_2_ respectively. Water repellence of soil was measured using the molarity of ethanol droplet test (MED^55^). Soil textural classes were defined using particle size analysis (PSA) as determined by sedimentation^56^ for a sub-set of sites (n = 324).

Particulate, humus and resistant organic C fractions reported in this study were derived from a mid-infrared (MIR) diffuse reflectance prediction following the calibration of a sub-set of 2 mm sieved soils which were dispersed and sieved to separate a >50 μm coarse fraction and a <50 μm fine fraction^57^. These fractions were analysed using ^13^C-NMR (nuclear magnetic resonance) spectroscopy to define the resistant organic C content of each soil based on the fraction of poly-aryl C present^58^). The particulate and humus fractions were calculated as the non-resistant material present in the coarse and fine fractions respectively^57^.

### Statistical analyses

Generalised additive mixed models were applied independently to explore the linear and non-linear relationships of SOC stocks (0–0.1, 0–0.3 m) with meta-physical and physio-chemical data within Western Australian soil groups^48^. The relevance of using Western Australian soil groups as a predictor in this analysis is based on prior research^51^ demonstrating a more localised soil classification system better estimated variance within specific measures such as SOC and ease of classification.

All analyses were conducted using R statistical software^59^. Soil organic C was checked for normality and where required a log transformation was applied. Linear regression determined SOC content (based on equivalent soil mass) in the 0 to 0.1 m and 0 to 0.3 m soil layers was strongly correlated (R^2^ = 0.93), with 62% of total SOC (0–0.3 m) on average located in the top 0.1 m of the soil. Given the strong influence of the surface 0.1 m, results have been presented for both the 0–0.1 m and 0–0.3 m soil layers separately where relevant. Based on the confidence in the mid-infrared prediction^57^ for measured particulate (P < 0.001; R^2^ = 0.82), humus (P < 0.001; R^2^ = 0.84) and resistant organic C (P < 0.001; R^2^ = 0.78) fractions in soil for Western Australian soils the relative importance of variables in determining fractions was also investigated.

A smoothing curve was fitted to each continuous variable to link with the SOC stocks^59^. Forwards variable selection was applied to remove insignificant variables. The model includes random effects to account for correlation among observations on the same sampling unit (region, farm, paddock and sample point), using an autoregressive structure with lag 1. Binary regression decision trees were built to repeatedly split the predictor space according to splitting rules based on predictor variables^60^. Here separate binary regression trees were applied to total SOC stock and the particulate, humus and resistant organic C fractions. These trees identify the important non-linear interactions between climate, soil and management variables. Trees were implemented using the recursive partitioning algorithm in R.

Principle component analysis (PCA) was also applied to investigate how the climate, soil and management variables were correlated. Only continuous variables were included in the analysis and all were first normalised. Since the soil classification variables are categorical these could not be included, however the percentage sand was included as a proxy. The 10 year rotational variable was converted into several variables (i.e. presence/absence of each cropping category). Exploratory analyses were conducted for individual variables of interest such as rainfall, soil pH and temperature where forward regression of all variables suggested high relative importance. Linear regression was applied to explore both the relationship between SOC, clay and water repellence as well as to average annual rainfall, while changes in SOC over all sites in Western Australia were examined using change point regression analysis in R (using the strucchange library in R) at each depth interval. Change point regression analysis was also applied to examine changes in each of mid-infrared prediction of particulate, humus and resistant organic C. These change points were confirmed using generalised additive mixed models to explore the linear or non-linear relationship of SOC with the variable of interest. Below and above each change point, a linear regression was applied to explore the relationship between SOC and pH. The model includes random effects to account for correlation among observations on the same sampling unit (region, farm, paddock and sample point), using an autoregressive structure with lag 1.

## Additional Information

**How to cite this article**: Hoyle, F. C. *et al.* Spatially governed climate factors dominate management in determining the quantity and distribution of soil organic carbon in dryland agricultural systems. *Sci. Rep.* **6**, 31468; doi: 10.1038/srep31468 (2016).

## Supplementary Material

Supplementary Information

## Figures and Tables

**Table 1:** Relative importance of parametric coefficients on variability in total soil organic carbon (t C ha^−1^) at (a) 0–0.1 m and (b) 0–0.3 m for 1348 sample points in Western Australia when including^a^ or excluding^b^ primary climate drivers such as average annual rainfall (Rain30y), daily temperature (Temp30y) and soil textural group (Supergroup).

All^a^		<473 mm^b^		473–600 mm^b^		>600 mm^b^	
**(a) 0–0.1 m**							
Rain30y	0.27	Supergroup	0.78	Skg_5y	0.32	Skg_5y	0.33
Rotation10y	0.18	Kkg_5y	0.09	Supergroup	0.32	pH	0.19
Temp30y	0.13	pH	0.08	Rotation10y	0.13	Pkg_5y	0.15
Skg_5y	0.07			pH	0.09	Rotation10y	0.15
pH	0.06			Nkg_5y	0.06	Stock	0.12
Stock	0.05						
*R-sq. adj.* (*sites*)	*0.72* (*n* = *966*)		*0.41* (*n* = *475*)		*0.54* (*n* = *306*)		*0.31* (*n* = *198*)
**(b) 0–0.3 m**							
**All**^**a**^		**<600 mm**^**b**^ **>17.2 °C**		**<600 mm**^**b**^**<17.2 °C**		**>600 mm**^**b**^ **All temperatures**	
Rain30y	0.29	Supergroup	0.33	Skg_5y	0.41	Skg_5y	0.37
Rotation10y	0.19	pH (CaCl_2_)	0.32	Supergroup	0.37	pH (CaCl_2_)	0.23
Temp30y	0.14	Rotation10y	0.20	Nkg_5y	0.13	Stock	0.17
Skg_5y	0.08	Kkg_5y	0.09	Rotation10y	0.07	Rotation10y	0.17
Stock	0.06						
Supergroup	0.05						
*R-sq.* (*adj*)	*0.79* (*n* = *973*)		*0.18* (*n* = *351*)		*0.51* (*n* = *442*)		*0.27* (*n* = *182*)

Average sulphur (Skg_5y), phosphorous (Pkg_5y), potassium (Kkg_5y) and nitrogen (Nkg_5y) fertiliser applications (kg ha^−1^) over the most recent five year history, rotation over the previous 10 years (Rotation10y), soil pH and the presence or absence of stock (Stock) is included. Non-significant variables (p > 0.05) are not reported in this table.

**Table 2:** The relationship between soil organic carbon and average annual temperature at 0–0.1 m and 0–0.3 m, below and above the average change point in annual temperature of 17.2 °C (n = 1348 sample points).

Depth	Temp30y	F-statistic	p-value	Regression	Adjusted R^2^
0–0.1 m	<17.2	470.1	<0.0001	118.09–5.62 × temperature	0.2584
0–0.1 m	>17.2	16.6	<0.0001	14.79–0.38 × temperature	0.0310
0–0.3 m	<17.2	452.9	<0.0001	177.56–8.33 × temperature	0.2518
0–0.3 m	>17.2	0.18	0.672	14.76–0.07 × temperature	0.0003

**Table 3:** Change point regression analysis for relationship between soil pH and fractions (particulate organic carbon, POC; humus organic carbon, HOC and resistant organic carbon, ROC; t C ha^−1^) for all areas analysed together (n = 1348 sample points).

Fraction	Depths	Change Point	F	p-value
Total SOC	All	4.61	74.9	<0.0001
	0.0–0.1 m	4.44	109.0	<0.0001
	0.1–0.2 m	4.09	270.1	<0.0001
	0.2–0.3 m	4.02	283.9	<0.0001
	0.2–0.3 m	7.98	14.6	0.014
POC	All	5.3	292.7	<0.0001
	0.0–0.1 m	5.5	252.7	<0.0001
	0.1–0.2 m	5.0	592.8	<0.0001
	0.2–0.3 m	5.2	906.6	<0.0001
HOC	All	5.3	102.9	<0.0001
	0.0–0.1 m	5.6	142.8	<0.0001
	0.1–0.2 m	4.3	91.3	<0.0001
	0.2–0.3 m	5.2	152.7	<0.0001
ROC	All	5.3	240.3	<0.0001
	0.0–0.1 m	5.6	208.5	<0.0001
	0.1–0.2 m	5.0	326.3	<0.0001
	0.2–0.3 m	5.2	794.3	<0.0001

Depth information is only presented where different from overall analysis.

**Table 4:** Relative importance of parametric coefficients on particulate (POC), humus (HOC) and resistant (ROC) organic carbon fractions (t C ha^−1^) in soil to 0–0.1 m (n = 1079) and 0–0.3 m (n = 973 sites) in Western Australia.

Soil depth (m)	POC		HOC		ROC	
0.0–0.1	Rain30y	0.25	Rain30y	0.21	Rain30y	0.24
	Supergroup	0.18	Temp30y	0.22	Temp30y	0.15
	Temp30y	0.17	Rotation	0.16	Rotation	0.14
	Rotation	0.12	Supergroup	0.08	Supergroup	0.13
	Stock	0.05	Stock	0.05	Stock	0.06
	pH	0.02	pH	0.01	pH	0.001
*R-sq. adj.* (n = 1079)		*0.82*		*0.75*		*0.79*
0.0–0.3	Supergroup	0.26	Supergroup	0.37	Supergroup	0.32
	pH (CaCl_2_)	0.14	pH (CaCl_2_)	0.13	pH (CaCl_2_)	0.20
	Rain30y	0.13	Temp30y	0.12	Rain30y	0.10
	Temp30y	0.12	Rain30y	0.09	Temp30y	0.08
			Skg_5y	0.06	Skg_5y	0.05
*R-sq. adj.* (n = 973)		*0.83*		*0.72*		*0.81*
