# Supplementary material for: Spatially governed climate factors dominate management in determining the quantity and distribution of soil organic carbon in dryland agricultural systems
Source: Sci Rep. 2016 Aug 17;6:31468. doi: 10.1038/srep31468 (PMC4987626; doi:10.1038/srep31468)
Supplement: Supplementary Information [file srep31468-s1.pdf]

Supplementary Information

Title: Spatially governed climate factors dominate management in determining the quantity and distribution of soil organic carbon in dryland agricultural systems

Running head: Primary drivers of soil organic carbon

Authors: Frances C Hoyle[AB], Rebecca A O'Leary[A], Daniel V Murphy[B]

Figures

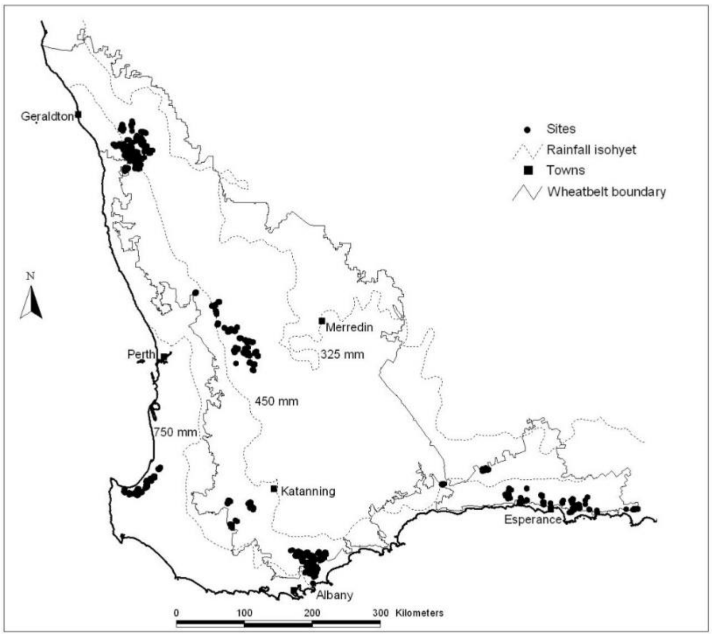

Supplementary Figure S1 Map of south-west Western Australia showing rainfall isohyets and location of sites sampled between 2010-2012 for soil organic carbon stocks (0-0.3 m; n=1160 sites). This image was generated using GeoMedia®, which is a software product owned by Intergraph Corporation doing business as Hexagon Geospatial. ©2011-2016 Hexagon AB and/or its subsidiaries and affiliates. (http://www.hexagongeospatial.com/products/producer-suite/geomedia).

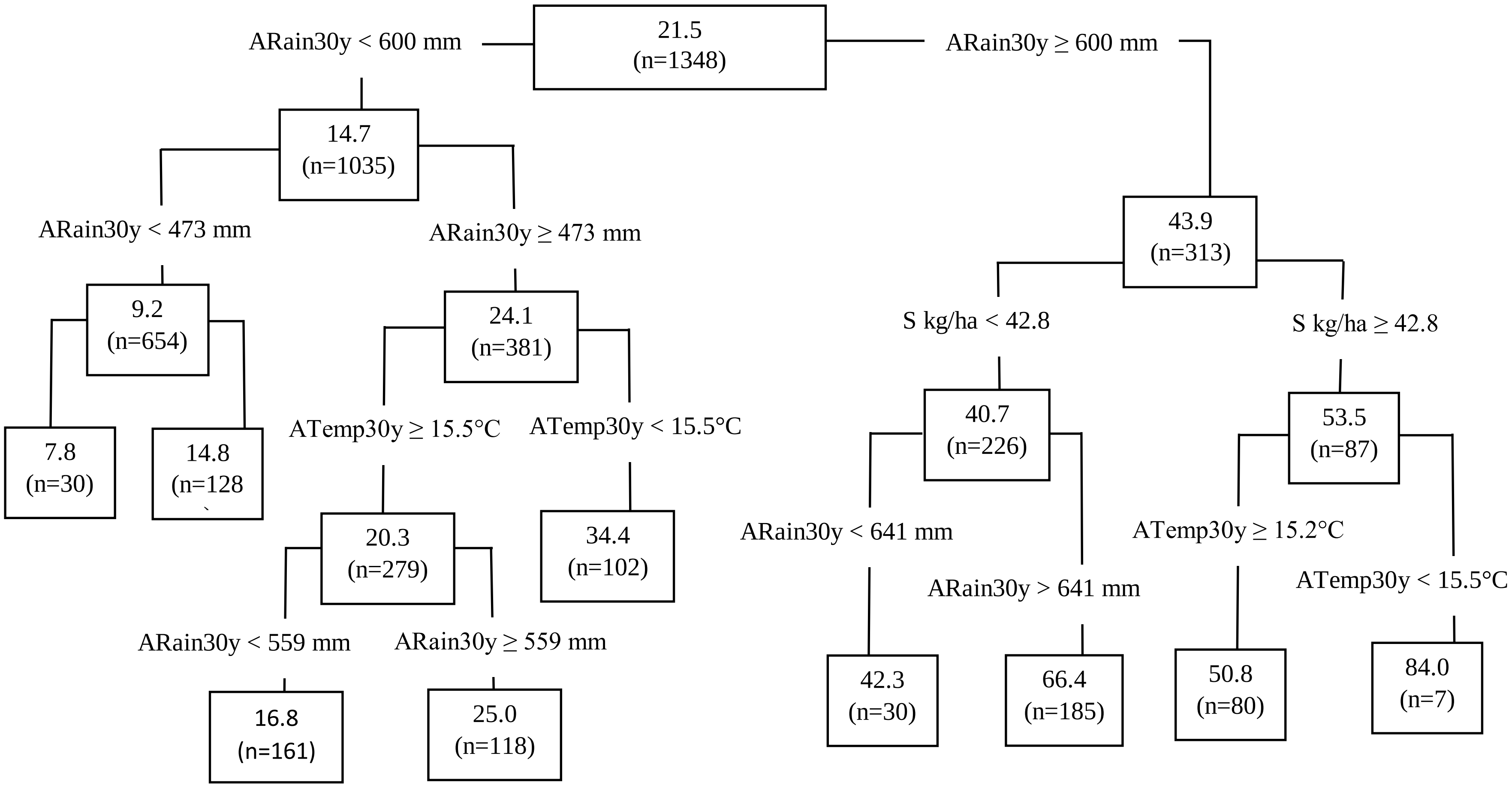

Supplementary Figure S2 Binary regression decision tree[60] for soil organic carbon (0-0.1 m, t C ha^{-1}, r^2=72.4%) identifying the important non-linear interactions between climate, soil and management variables. Trees were implemented using the recursive partitioning algorithm in R[59].

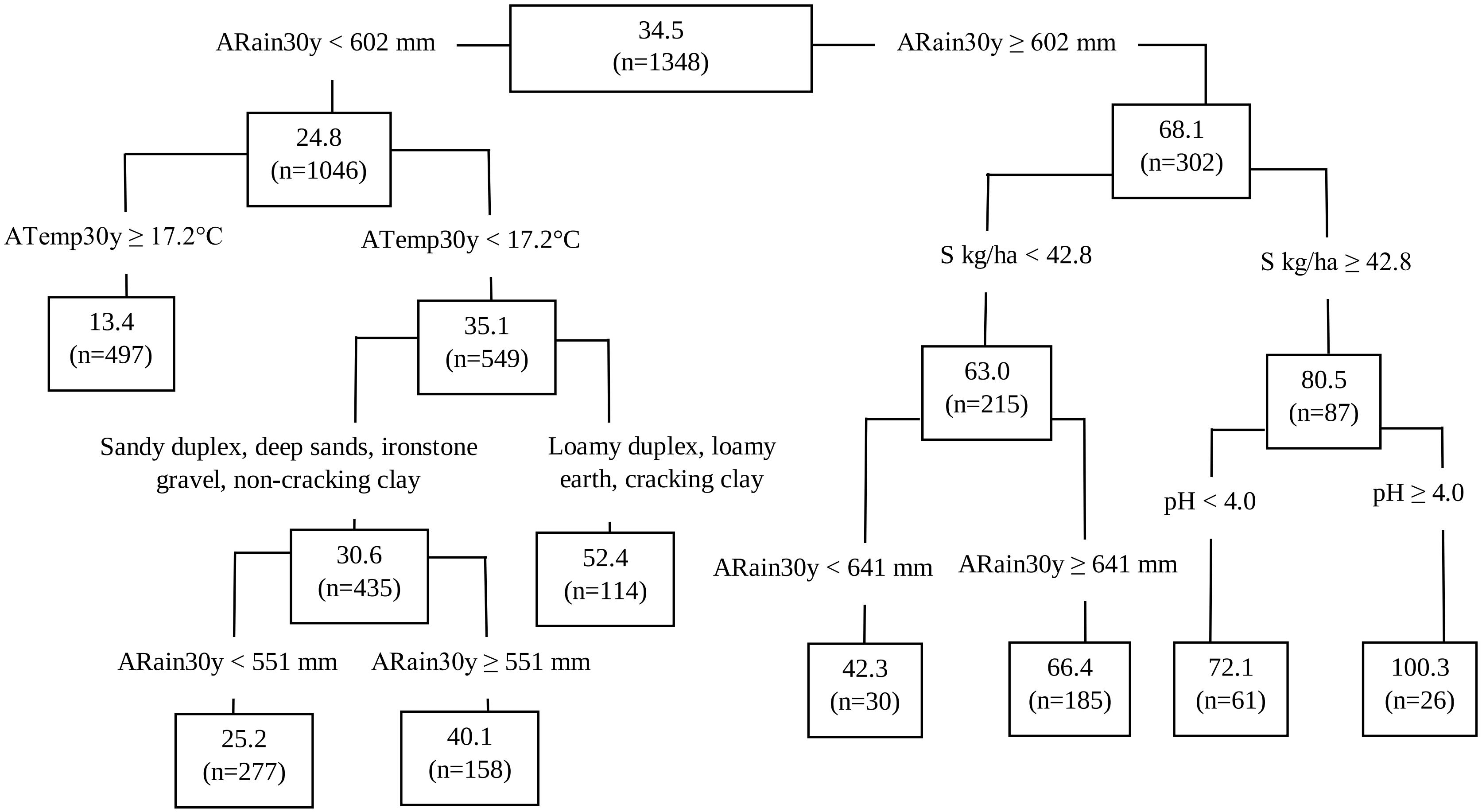

Supplementary Figure S3 Binary regression decision tree[60] for soil organic carbon (0-0.3 m, t C ha^{-1}, r^2=70.2%) identifying the important non-linear interactions between climate, soil and management variables. Trees were implemented using the recursive partitioning algorithm in R[59].

Tables

Supplementary Table S1 Land use sequence (prior 10 year history) describing dominant Australian Soil Classification[52] (and non-dominant classifications) within each soil textural class[48] (0-0.3 m; n=1160 sites).

Soil textural class	Dominant Australian Soil Classification	Continuous cropping (n=191)	Crop Dominant (n=233)	Mixed cropping (n=174)	Pasture Dominant (n=171)	Continuous Pasture (n=391)
Cracking clay (n=14)	Vertosol	0	13	0	1	0
Deep sand (n=672)	Tenosol (Podosol, Chromosol, Sodosol)	96	138	91	112	235
Ironstone gravel (n=23)	Chromosol (Sodosol, Tenosol)	2	13	0	3	5
Loamy duplex (n=94)	Sodosol (Kurosol, Chromosol)	3	19	28	1	43
Loamy earth (n=30)	Kandosol (Tenosol)	14	5	5	2	4
Non-cracking clay (n=6)	Dermosol	2	3	1	0	0
Sandy duplex (n=262)	Sodosol (Chromosol, Kurosol, Kandosol)	41	34	43	51	93
Sandy earth (n=59)	Chromosol (Sodosol, Tenosol)	33	8	6	1	11

Supplementary Table S2 Mean and standard error (minimum, maximum) for soil properties measured across different soil textural classes at 0-0.1, 0.1-0.2 and 0.2-0.3 m depth intervals at all sample points (n=1348). MED = Molarity of ethanol drop test[55] for water repellence rating where a score of 0.0 is no detectable water repellence, ≤1.0 is low, 1.2-2.2 is moderate and ≥2.4 is severely water repellent. NA = not available.

Soil textural class	Lower depth (m)	Bulk density (g cm^3)	Clay (%)	Sand (%)	Electrical conductivity (µS cm^{-1})	MED (mol.)	Soil pH ($CaCl_2$)
Ironstone gravel	0.1	1.51±0.02 (1.3, 1.6)	4.7±1.0 (0.7, 9.3)	92.8±1.2 (86.1, 97.1)	107±10 (50, 235)	1.6±0.2 (0, 3.6)	4.8±0.1 (4.2, 5.6)
	0.2	1.70±0.03 (1.5, 2.1)	2.8±0.3 (2.4, 3.1)	95.7±0.1 (95.6, 95.8)	45±3 (12, 99)	0.3±0.1 (0, 2.4)	4.5±0.1 (4.0, 5.4)
	0.3	1.69±0.04 (1.4, 2.3)	2.1±0.4 (1.6, 2.6)	96.3±0.3 (95.8, 96.7)	43±3 (17, 71)	0.1±0.1 (0, 1.2)	4.6±0.1 (4.1, 5.2)
Loamy duplexes	0.1	1.42±0.01 (1.2, 1.7)	12.1±3.8 (3.5, 19.6)	82.7±4.8 (72.6, 93.1)	138±9 (20, 464)	0.1± 0.0 (0, 1.2)	5.4±0.1 (4.1, 8.0)
	0.2	1.66±0.01 (1.4, 2.0)	23.3±5.7 (10.0, 33.8)	70.4±7.3 (56.5, 87.0)	112±14 (27, 1006)	0.0	5.4±0.1 (4.1, 8.3)
	0.3	1.64±0.02 (1.3, 2.5)	32.5±4.3 (24.2, 42.3)	61.0±6.15 (46.8, 72.5)	131±17 (22, 1075)	0.0	5.6±0.1 (4.0, 8.5)
Loamy earths	0.1	1.52±0.01 (1.1, 1.7)	6.3±0.3 (3.9, 10.2)	90.2±1.3 (87.0, 92.0)	112±18 (24, 1050)	0.2±0.1 (0, 2.8)	5.2±0.1 (4.2, 8.0)
	0.2	1.67±0.01 (1.2, 1.9)	11.9±1.7 (7.8, 14.4)	84.7±1.6 (82.3, 88.6)	170±69 (21, 4104)	0.0±0.0 (0, 1.2)	5.0±0.1 (4.0, 8.2)
	0.3	1.64±0.02 (1.1, 2.0)	14.8±2.2 (10.0, 19.4)	81.9±2.0 (77.9, 86.5)	104±32 (13, 1458)	0.0±0.0 (0, 0.2)	5.4±0.1 (4.0, 8.5)
Cracking clays	0.1	1.31±0.05 (1.2, 1.7)	33.1±1.4 (21.2, 40.0)	NA	254±13 (160, 362)	0.0	7.8±0.1 (6.7, 8.1)
	0.2	1.39±0.04 (1.2, 1.8)	35.2±1.3 (21.3, 44.4)	NA	313±14 (195, 417)	0.0	8.0±0.1 (7.2, 8.3)
	0.3	1.40±0.04 (1.3, 1.8)	28.9±1.1 (21.0, 34.8)	NA	424±22 (288, 580)	0.0	8.2±0.1 (7.5, 8.6)
Non-cracking clays	0.1	1.38±0.03 (1.2, 1.7)	26.5±1.8 (23.6, 32.4)	60.7±4.1 (48.0, 71.1)	151±15 (63, 320)	0.0	6.3±0.3 (4.3, 7.6)
	0.2	1.52±0.03 (1.3, 1.8)	31.0±3.2 (23.0, 39.1)	55.1±3.3 (49.7, 66.4)	270±83 (66, 1889)	0.0	6.6±0.3 (4.3, 7.8)
	0.3	1.46±0.03 (1.2, 1.8)	28.9±7.9 (1.6, 41.3)	55.9±9.8 (37.2, 88.8)	238±44 (31, 946)	0.0	6.9±0.2 (4.9, 8.1)
Deep sands	0.1	1.41±0.00 (0.9, 1.8)	3.7±0.2 (0.4, 12.2)	94.8±0.3 (75.8, 99.5)	117±5 (13, 1400)	2.0±0.1 (0, 4.8)	5.0±0.0 (3.5, 7.3)
	0.2	1.57±0.00 (1.2, 2.4)	2.3±0.2 (0.2, 6.7)	96.3±0.3 (91.4, 99.1)	52±3 (9, 1977)	1.2±0.1 (0, 4.8)	4.4±0.0 (3.2, 6.9)
	0.3	1.52±0.01 (1.1, 2.0)	1.9±0.25 (0.2, 7.6)	96.7±0.4 (90.6, 99.3)	35±1 (2, 390)	0.8±0.1 (0, 4.8)	4.3±0.0 (3.2, 7.1)
Sandy duplexes	0.1	1.41±0.01 (0.8, 1.7)	5.6±0.2 (0.7, 20.2)	91.7±0.3 (70.9, 98.2)	136±6 (27, 95)	2.0±0.1 (0, 4.2)	4.8±0.0 (3.8, 6.8)
	0.2	1.65±0.01 (1.4, 2.2)	12.5±1.2 (2.1, 30.5)	83.6±1.3 (66.3, 96.7)	71±4 (12, 578)	0.2±0.0 (0, 2.6)	4.6±0.0 (3.8, 8.2)
	0.3	1.59±0.01 (1.2, 2.2)	20.1±1.6 (0.7, 45.5)	76.3±1.6 (53.0, 97.3)	87±6 (4, 743)	0.0±0.0 (0, 1.4)	4.9±0.0 (3.9, 8.5)
Sandy earths	0.1	1.46±0.01 (0.9, 1.6)	3.6±0.3 (1.6, 8.8)	94.4±0.3 (90.4, 96.7)	143±13 (30, 925)	0.8±0.1 (0, 4.6)	5.2±0.0 (4.0, 6.4)
	0.2	1.65±0.01 (1.2, 1.8)	3.1±0.3 (2.4, 4.3)	94.8±0.4 (93.3, 96.4)	68±15 (15, 1806)	0.2±0.1 (0, 3.2)	4.4±0.0 (3.3, 5.3)
	0.3	1.61±0.01 (1.4, 2.3)	3.9±0.7 (1.8, 7.2)	94.2±0.9 (90.0, 96.6)	37±4 (2, 320)	0.0±0.0 (0, 3.2)	4.3±0.0 (3.8, 4.9)

Supplementary Table S3 Mean and standard error (minimum, maximum) for total soil organic carbon (SOC; Elementar) , and particulate (POC), humus (HOC) and resistant (ROC) carbon fractions determined by mid-infrared spectroscopy[57] for different soil textural classes at 0-0.1, 0.1-0.2, 0.2-0.3 and 0.0-0.3 m depth intervals (n=1348 sites).

Soil textural class	Lower depth (m)	SOC (t C ha^{-1})	POC (t C ha^{-1})	HOC (t C ha^{-1})	ROC (t C ha^{-1})
Ironstone gravel	0.1	16.5±1.9 (5.2, 45.48)	4.4±0.6 (1.4, 13.0)	5.3±0.8 (1.4, 13.5)	3.5±0.4 (1.4, 8.0)
	0.2	4.9±0.6 (1.1, 13.6)	1.1±0.2 (0.2, 3.0)	2.2±0.4 (0.7, 7.8)	1.4±0.1 (0.7, 2.7)
	0.3	3.1±0.4 (1.2, 6.5)	0.6±0.1 (0.1, 1.4)	2.3±0.4 (0.7, 7.5)	1.1±0.1 (0.7, 1.9)
	0.0-0.3	24.5±2.7 (8.6, 65.2)	6.1±0.8 (2.7, 17.2)	9.9±1.5 (3.1, 26.6)	6.0±0.5 (3.1, 12.1)
Loamy duplex	0.1	29.6±1.4 (4.3, 62.1)	6.5±0.4 (0.3, 22.1)	15.5±0.7 (2.4, 29.6)	7.9±0.3 (1.5, 18.5)
	0.2	11.4±0.6 (2.5, 35.0)	2.5±0.4 (0.0, 19.7)	9.4±0.5 (1.1, 26.3)	4.1±0.3 (0.9, 18.2)
	0.3	8.4±0.7 (2.0, 43.7)	3.2±0.7 (0.0, 34.2)	9.5±0.6 (2.0, 35.7)	3.5±0.4 (0.4, 22.3)
	0.0-0.3	49.8±2.2 (10.3, 140.4)	12.4±1.3 (0.6, 59)	35.2±1.5 (6.8, 80.1)	15.6±1 (3, 53.2)
Loamy earth	0.1	9.4±1.3 (3.3, 45.5)	2.1±0.6 (0.1, 17.3)	4.6±0.6 (1.8, 16.8)	2.9±0.4 (1.0, 14.7)
	0.2	5.4±0.5 (2.1, 15.7)	1.4±0.6 (0.0, 19.6)	4.3±0.5 (1.5, 18.8)	2.3±0.4 (0.5, 16.4)
	0.3	4.2±0.6 (1.4, 26.8)	1.4±0.7 (0.0, 22.8)	4.2±0.6 (1.4, 23.9)	2.1±0.5 (0.4, 19.3)
	0.0-0.3	19.2±2.1 (7.2, 68)	4.9±1.8 (0.1, 56.7)	13.5±1.5 (4.7, 59.5)	7.4±1.4 (2.4, 50.3)
Cracking clay	0.1	15.9±0.5 (14.1, 20.7)	10.5±1.0 (5.0, 18.3)	11.3±0.8 (7.4, 16.5)	9.0±0.5 (6.2, 13.2)
	0.2	12.9±0.6 (6.0, 15.3)	16.4±2.1 (3.3, 28.6)	13.8±0.9 (9.1, 20.6)	11.3±1.1 (4.5, 17.8)
	0.3	16.2±1.8 (3.8, 23.2)	26.0±3.7 (2.3, 43.9)	19.4±1.8 (8.0, 28.5)	16.2±1.9 (4.1, 25.3)
	0.0-0.3	45.0±2.1 (26.2, 54.7)	52.9±6.2 (11.3, 76.7)	44.6±2.2 (30.3, 55.4)	36.5±3.1 (15.4, 49.4)
Non-cracking clay	0.1	10.8±1.0 (6.3, 23.1)	2.1±0.6 (0.5, 10.8)	7.7±1.0 (4.2, 21.3)	3.8±0.5 (1.0, 9.6)
	0.2	6.8±0.6 (3.5, 13.1)	2.4±0.7 (0.1, 10.6)	6.6±0.8 (4.0, 18.3)	3.3±0.5 (0.4, 9.1)
	0.3	6.1±0.8 (2.3, 17.2)	3.6±1.4 (0.0, 24.6)	6.9±1.0 (3.3, 20.4)	3.5±0.6 (0.2, 9.7)
	0.0-0.3	23.7±1.9 (12.1, 43.8)	8.1±2.4 (0.7, 37.9)	21.3±2.8 (12.9, 60)	10.5±1.5 (1.7, 28.5)
Deep sand	0.1	20.3±0.7 (1.5, 136.3)	5.3±0.2 (0.3, 34.6)	5.9±0.2 (0.7, 31.9)	4.0±0.1 (0.7, 20.3)
	0.2	8.0±0.3 (1.1, 52.2)	1.8±0.1 (0.0, 14.6)	2.5±0.1 (0.0, 13.3)	1.8±0.0 (0.3, 8.8)
	0.3	4.3±0.2 (0.6, 34.9)	0.9±0.0 (0.0, 8.9)	1.6±0.0 (0.2, 11.4)	1.1±0.0 (0.1, 7.1)
	0.0-0.3	32.6±1.1 (4.2, 208.5)	8.0±0.3 (0.6, 58.1)	10.1±0.3 (1.6, 54.1)	7.0±0.2 (1.8, 34.7)

Sandy duplex	0.1	25.3±1.0 (3.3, 147.6)	8.0±0.3 (0.2, 36.7)	9.6±0.4 (0.9, 33.6)	5.5±0.2 (0.9, 18.9)
	0.2	7.7±0.3 (1.3, 66.7)	1.6±0.1 (0.0, 20.1)	4.5±0.2 (0.5, 23.9)	2.1±0.1 (0.5, 12.6)
	0.3	5.3±0.2 (0.7, 18.1)	1.0±0.1 (0.0, 13.1)	4.4±0.2 (0.4, 15.1)	1.5±0.1 (0.1, 12.3)
	0.0-0.3	38.5±1.5 (5.4, 176)	10.6±0.4 (0.3, 46.3)	18.8±0.7 (2.2, 60.8)	9.1±0.3 (2, 34)
Sandy earth	0.1	16.4±1.7 (3.9, 94.6)	4.0±0.6 (0.23, 29.6)	5.5±0.5 (1.5, 22.3)	3.5±0.3 (1.1, 13.3)
	0.2	5.1±0.5 (0.0, 25.6)	0.8±0.1 (0.1, 6.1)	2.6±0.1 (0.7, 9.6)	1.5±0.1 (0.6, 5.8)
	0.3	2.9±0.2 (1.0, 10.9)	0.3±0.0 (0.0, 2.7)	2.1±0.1 (0.4, 5.5)	1.1±0.0 (0.5, 3.0)
	0.0-0.3	24.6±2.3 (6.8, 122.3)	5.1±0.7 (0.4, 38.4)	10.5±0.7 (3.1, 30.4)	6.2±0.4 (2.6, 19.8)
